# Attention to advertising and memory for brands under alcohol intoxication

**DOI:** 10.3389/fpsyg.2014.00212

**Published:** 2014-03-25

**Authors:** Jacob L. Orquin, Heine B. Jeppesen, Joachim Scholderer, Curtis Haugtvedt

**Affiliations:** ^1^Department of Business Administration – MAPP, Aarhus UniversityAarhus C, Denmark; ^2^Fisher College of Business, Ohio State UniversityColumbus, OH, USA

**Keywords:** advertising, eye movements, alcohol intoxication, memory, brand recall

## Abstract

In an attempt to discover new possibilities for advertising in uncluttered environments marketers have recently begun using ambient advertising in, for instance, bars and pubs. However, advertising in such licensed premises have to deal with the fact that many consumers are under the influence of alcohol while viewing the ad. This paper examines the effect of alcohol intoxication on attention to and memory for advertisements in two experiments. Study 1 used a forced exposure manipulation and revealed increased attention to logos under alcohol intoxication consistent with the psychopharmacological prediction that alcohol intoxication narrows attention to the more salient features in the visual environment. Study 2 used a voluntary exposure manipulation in which ads were embedded in a magazine. The experiment revealed that alcohol intoxication reduces voluntary attention to ads and leads to a significant reduction in memory for the viewed ads. In popular terms consuming one or two beers reduces brand recall from 40 to 36% while being heavily intoxicated further reduces brand recall to 17%.

## INTRODUCTION

Our world is cluttered with visual information. Since our attention capacity is limited, we can only process objects we encounter during a day to varying degrees of depth – sometimes processing extensively and other times at a very superficial level. A working assumption of most marketers is that more extensive processing should result in more positive outcomes for the advertised object or issue. Perhaps as a consequence of increased advertising clutter (Pieters et al., 2007) or consumer antagonism to traditional advertising (Jensen et al., 2014) a variety of non-traditional advertising strategies have been suggested as ways to get consumers to pay attention to and process advertisements more extensively. Many strategies have involved attempts to increase the salience of the advertising stimulus by placing it in uncluttered environments. One such strategy is to advertise in licensed premises, using different types of media such as restroom advertisements, promotional beer mats, and pub TV systems. The low density of advertisements in licensed premises could suggest that such a strategy might indeed be successful (Pieters et al., 2007). However, proponents of this strategy have to contend with the fact that customers in licensed premises consume alcohol. Research on the psychopharmacological effects of alcohol has demonstrated serious impairments at perceptual and post-perceptual stages of information processing, including impairments of attention functions such as object recognition (Maylor et al., 1987), allocation of resources to stimulus analysis and response selection (Pickworth et al., 1997; De Cesarei et al., 2006) and conceptual processing functions such as encoding and elaboration (Hashtroudi et al., 1983; Saults et al., 2007; Söderlund et al., 2007). In general, these impairments result in (a) a narrowing of visual attention to the most salient features in a complex stimulus (b) shallow processing of conceptual information and (c) memory loss.

In the context of advertising exposure under the influence of alcohol, it can therefore be expected that ad elements which are predominantly processed by perceptual mechanisms, such as logos and images, will have a selective advantage over ad elements that are predominantly processed by conceptual mechanisms, such as headlines and text blocks. It furthermore seems plausible that alcohol intoxication will have a detrimental effect on brand recall although the strength of such an effect is probably moderated by attention to important ad elements like the logo. To the best of our knowledge, no research on the influence of alcohol consumption and reactions to advertising has been reported. The current research is an exploration of how various levels of alcohol might influence perception of advertising messages. While the current research focuses on traditional product advertising, the procedures and results of this research may have implications for the way individuals react to various public and personal safety messages under varying degrees of alcohol consumption.

## STUDY 1

Study 1 addressed the question of how alcohol intoxication affects visual attention to ad elements. As suggested above, psychopharmacological effects of alcohol intoxication such as a narrowing of the attention span to salient stimuli might translate well into advertising perception to mean that intoxicated consumers will focus more on perceptual ad elements like the logo or the image. However, the degree of attention to perceptual versus conceptual ad elements is likely to depend on the balance between perceptual and conceptual elements in the visual scene (Wedel and Pieters, 2007; Orquin et al., 2013a; Peschel and Orquin, 2013). To test whether the effect of alcohol on attention to advertising depends on the balance between perceptual and conceptual elements, we conducted an eye tracking experiment manipulating the perceptual and conceptual load of advertisements. Eye tracking provides an objective measure of eye movements which is a reliable indicator of overt visual attention (Orquin and Mueller Loose, 2013).

### METHODS

#### Participants

Thirty six undergraduate and graduate business students with specializations other than marketing or corporate communications were recruited on campus for participation in the study. Their mean age was 23.87 years (SD = 1.83), 36% were female.

#### Experimental design

Two factors of the advertising stimuli were varied: (1) brand (12 levels, representing consumer goods, services, and corporate brands) and (2) perceptual and conceptual load (three levels: high perceptual load with a dominance of pictorial elements, high perceptual and conceptual load with a balance between pictorial and text elements, and high conceptual load with a dominance of text elements). Pretesting was used to determine these levels. The two factors were completely crossed in the master design, resulting in 36 stimuli. The design was then blocked in such a way that each participant was exposed to all twelve levels of the first factor, brand, and at equal proportions of the levels of the second factor, perceptual and conceptual load.

#### Materials and measures

The 36 experimental ads were developed using a graphic design software and were all based on existing market stimuli. All ads used color and were displayed in a similar size on a 21 inch color screen.

For ethical reasons we decided not to manipulate the blood alcohol concentration (BAC) of our participants. Instead we measured the BAC levels of already sober and intoxicated participants using a digital breathalyzer. BAC levels ranged between 0% (sober) and 0.164% (heavily intoxicated), with a mean level of 0.056% (SD = 0.054).

Measures of visual attention were obtained by means of eye tracking (Tobii 2150, frame rate: 50 frames per second). Three measures of eye movements were extracted from the eye tracker logs for each major ad element (headline, logo, image, text) in each ad: time to first fixation on the ad element, number of fixations before the first fixation on the element occurred (both measured from stimulus onset), and total fixation time to each ad element.

#### Procedure

All participants were recruited in the university student club in the late afternoon and evening hours and accompanied to the lab facilities by the experimenter. Before the experiment started, each participant’s BAC was tested using a digital breathalyzer. Participants were positioned in front of the eye tracker and after calibration and a series of training stimuli each participant was randomly assigned to a block of 12 advertising stimuli. Each stimulus was presented for 10 sec thus creating a competition for attention among the ad elements (Orquin and Scholderer, 2011). After the experiment participants were thanked and accompanied back to the student club.

### RESULTS

To test the hypothesis that alcohol intoxication influences the salience of logos we analyzed the effect of BAC on eye movements to the four major ad elements (headline, logo, image, text) by means of Cox regression. The models were specified in such a way that the effects of BAC were estimated separately within levels perceptual and conceptual load, controlling for brand and stratified by participant. Instances where a participant had not fixated on an ad element were defined as censored events. Likelihood ratio tests of the significance of the alcohol effect are reported in **Table 1**.

**Table 1:** Effect of blood alcohol concentration on the visual salience of ad elements (headline, logo, image, text) under different levels of perceptual and conceptual load.

		Dependent variable					
		Fixations before			Time to first fixation		
Perceptual and conceptual load	Ad element	LR χ^2^	df	*p*	LR χ^2^	df	*p*
High perceptual load (dominance of pictorial elements)	Headline	1.116	1	0.291	1.966	1	0.161
	Logo	5.841	1	0.016	0.010	1	0.921
	Image	0.020	1	0.886	3.516	1	0.061
High perceptual and conceptual load (balance between pictorial and text elements)	Headline	0.029	1	0.865	0.050	1	0.822
	Logo	9.541	1	0.002	0.721	1	0.396
	Image	0.800	1	0.371	1.016	1	0.313
	Text block	1.700	1	0.192	0.251	1	0.616
High conceptual load (dominance of text elements)	Headline	0.942	1	0.332	3.815	1	0.051
	Logo	6.574	1	0.010	1.939	1	0.164
	Text block	2.820	1	0.093	5.041	1	0.025

**FIGURE 1:**
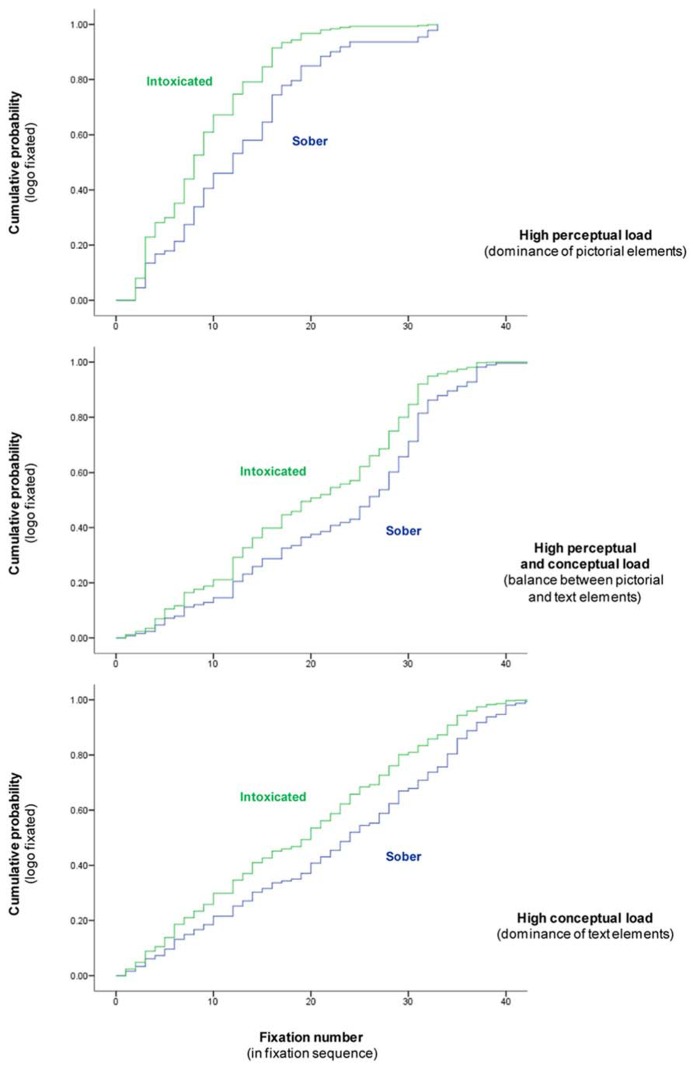
**Cumulative probabilities of fixating the logo for intoxicated participants (mean BAC = 0.102%, SD = 0.032) relative to sober participants**.

The analysis revealed that increased alcohol intoxication led to a significant amplification of the salience of the logo. A general reduction was observed in the number of fixations to other ad elements that occurred before participants fixated on the logo for the first time. Cumulative probabilities for the first fixation on the logo are plotted in **Figure 1**.

The effect of alcohol intoxication on salience of the logo was not moderated by the degree of perceptual and conceptual load in the advertisements. The amplification of salience also occurred in the high perceptual load condition, ruling out the alternative explanation that the effect could have been an artifact, caused by a reduction in conceptual information processing.

This is not to say that impairments of conceptual processing did not occur. In the high conceptual load condition, alcohol intoxication led to significant increases in the *time* before the first fixations on headline and text block occurred (see **Table 1**), suggesting a slowing-down of the conceptual information processing mechanisms. Furthermore, additional analyses revealed that alcohol intoxication led to significant decreases in the accumulated number of times participants fixated on the headline (Wald χ^2^[1] = 9.313, *p* = 0.002) and text block (Wald χ^2^[1] = 11.094, *p* = 0.001). No such effects were found for the visual elements, logo and image.

**Table 2** provides an overview of the effect of alcohol intoxication on eye movements to ad elements for three levels of alcohol intoxication: sober (BAC = 0%), intoxicated (BAC > 0% and ≤ 0.02%), and heavily intoxicated (BAC > 0.02%). The table contains three metrics the first of which is *fixation likelihood* which is the probability that a participant fixates an ad element, the second is *total fixation time* which is the average time participants spent viewing ad elements taking into account both the number and duration of fixations. The total fixation time is not conditional on participants having fixated the ad element. Trials in which a participant did not fixate the ad element are counted as zero total fixation time. The third metric, *fixations before*, is the average number of fixations that participants have to other ad elements before fixating the target element.

**Table 2:** Fixation likelihood, total fixation time and fixations before to the four add elements according to three levels of intoxication: sober (BAC = 0%), intoxicated (BAC ≤ 0.02%), heavily intoxicated (BAC ≤ 0.169%).

	Sober	Intoxicated	Heavily intoxticated
**Fixation likelihood**
Logo	0.73	0.67	0.74
Heading	0.97	0.92	0.88
Text	1.00	1.00	0.98
Image	1.00	0.99	0.98
**Total fixation time**
Logo	0.77	0.65	0.88
Heading	1.95	1.66	1.55
Text	6.36	5.89	5.56
Image	4.68	3.99	4.29
**Fixations before**
Logo	13.76	13.94	10.75
Heading	3.24	3.79	3.72
Text	3.32	3.16	3.08
Image	0.61	0.78	1.47

### DISCUSSION

The aim of the first study was to assess how visual attention to advertisements may be affected by alcohol intoxication. Based on established psychopharmacological findings, we hypothesized that the salience of the perceptual elements in complex advertisements would be selectively increased under conditions of alcohol intoxication, whereas the processing of conceptual information would be impaired. The results support our hypothesis, but in a more specific manner than originally expected: the selective increase in visual salience was only observed for logos (either brand or corporate) but not for other pictorial elements such as representations of products or human models. Additionally, we found the increased salience of logos was reflected in fixations before but not in time to first fixation. The only difference between the two metrics is that time to first fixation take the duration of individual fixations into account. Finding an effect for fixations before but not for time to first fixation therefore suggests that although intoxicated participants had fewer fixations before fixating the logo the duration of their fixations were longer than for sober participants. This interpretation seems consistent with the general finding that alcohol intoxication slows down cognitive processing.

The results suggest that “reminder” advertisements, primarily intended to increase the accessibility of the brand in the mind of the customer, will be effective in environments that involve the consumption of alcohol. Advertisements that intend to persuade, on the other hand, are likely to suffer.

Although the results confirm and extend psychopharmacological findings in the area of advertisement perception the interpretation may be limited due to the use of forced exposure to advertising stimuli. In Study 2 we address this issue by employing a voluntary exposure paradigm in which participants voluntarily fixate the advertising stimuli.

## STUDY 2

Study 2 examined the effect of alcohol intoxication on the distribution of attention to ads and ad elements as well as brand recall in a voluntary ad exposure paradigm. Whereas Study 1 used a forced exposure paradigm, Study 2 employed a procedure that more realistically simulated voluntary attention to ads in real life situations. The ads were embedded in a consumer magazine consistent with previous eye tracking research on voluntary attention to ads (Pieters et al., 2002, 2007; Pieters and Wedel, 2004). Such a procedure minimizes demand characteristics and furthermore allows assessment of whether alcohol intoxication has an influence on overall attention to ads. Study 2 used the same experimental design as Study 1 except that all experimental stimuli were embedded in a magazine.

### METHODS

#### Participants

Thirty six undergraduate and graduate business students with specializations other than marketing or corporate communications were recruited on campus for participation in the experiment. The mean age was 22.97 years (SD = 1.73), 44.5% were female.

#### Experimental design

The experimental design was identical to that in Study 1 manipulating the conceptual and perceptual load of ads for 12 different brands. The 36 experimental ads were blocked in three groups and inserted in a consumer magazine.

#### Materials and measures

The experimental stimuli consisted of 36 ads identical to those in Study 1. The ads were embedded in a consumer magazine with an even distribution of ad compositions and in a fixed order resulting in three different versions of the magazine. Each ad occupied an entire page in the magazine. As in Study 1 measures of BAC were obtained using a digital breathalyzer. BAC levels ranged from 0 to 0.169% (heavily intoxicated) with a mean level of 0.55% (SD = 0.53). Eye movements were recorded on the same eye tracker as in Study 1 and identical eye movement metrics were extracted from the log. Additionally, measures of brand recall were obtained from participants using a cued recall procedure. Brand recall was measured one day after the laboratory test.

#### Procedure

All participants were recruited in the school’s student club in the late afternoon and evening hours. Participants were accompanied to the lab facilities by the experimenter. Before the experiment started, each participant’s BAC was measured using a digital breathalyzer. Participants were positioned in front of the eye tracker and after the individual calibration of the eye tracker, the participants were randomly assigned to one of the three experimental blocks. Participants were informed that they could browse through the magazine at their own pace and the test ended when the participants reached the final page in the magazine. The magazine was presented one page at a time with each ad occupying an entire page. Participants were not informed about the purpose of the experiment. After the experiment participants were thanked and accompanied back to the student club. The day after the eye tracking study each participant received a questionnaire measuring cued brand recall. Sixty-four percent of the participants replied to the brand recall questionnaire.

### RESULTS

The first step in the analysis addressed the question of whether alcohol intoxication had any effect on advertising attention capture (whether the ad or ad element was fixated or not). The analysis was carried out by means of a generalized estimating equation with a logit link function and a binomial response distribution using attention capture as dependent variable and ad element (logo, headline, text, image, entire ad), ad version (perceptual load, conceptual load, mixed conceptual and perceptual load), and BAC as independent variables in a full factorial design. The results are reported in **Table 3** below.

**Table 3:** Effects of ad version, ad element and blood alcohol concentration on attention capture.

Effect	Wald Chi-Square	df	Significance	Goodness-of-fit
Intercept	163.936	1	0.000	QICC = 1378.757
Ad version	23.717	2	0.000		
Ad element	150.785	4	0.000		
BAC	9.562	1	0.002		
Ad version × Ad element	42.664	6	0.000		
Ad version × BAC	9.983	2	0.007		
Ad element × BAC	15.575	4	0.004		
Ad version × Ad element × BAC	19.447	6	0.003		

In order to interpret the results we extracted descriptive statistics for the effect of alcohol intoxication on attention capture to ad elements. Similar to Study 1, participants were grouped into three levels of alcohol intoxication: sober (BAC = 0%), intoxicated (BAC > 0% and ≤0.02%), and heavily intoxicated (BAC > 0.02%). The descriptive statistics are shown in **Table 4**.

**Table 4:** Fixation likelihood, total fixation time and fixations before to the four add elements and the entire ad according to three levels of intoxication: sober (BAC = 0%), intoxicated (BAC > 0% and ≤ 0.02%), heavily intoxicated (BAC > 0.02%).

	Sober	Intoxicated	Heavily intoxticated
**Fixation likelihood**				
Logo	0.56	0.57	0.27
Heading	0.90	0.93	0.76
Text	0.88	0.92	0.72
Image	0.93	0.94	0.86
Entire add	1.00	0.99	0.95
**Total fixation time**			
Logo	0.31	0.28	0.31
Heading	0.24	0.23	0.28
Text	0.35	0.35	0.36
Image	0.27	0.29	0.28
Entire add	0.25	0.26	0.27
**Fixations before**			
Logo	31.34	27.84	33.96
Heading	15.64	11.52	8.65
Text	23.10	24.81	16.91
Image	10.21	12.35	7.91
Entire add	6.44	4.26	4.87

It is clear from **Table 4** that alcohol intoxication has a negative effect on attention capture for all ad elements including the ad itself. The decrement in attention capture is particularly strong for logos, which is surprising given the results of Study 1.

**FIGURE 2:**
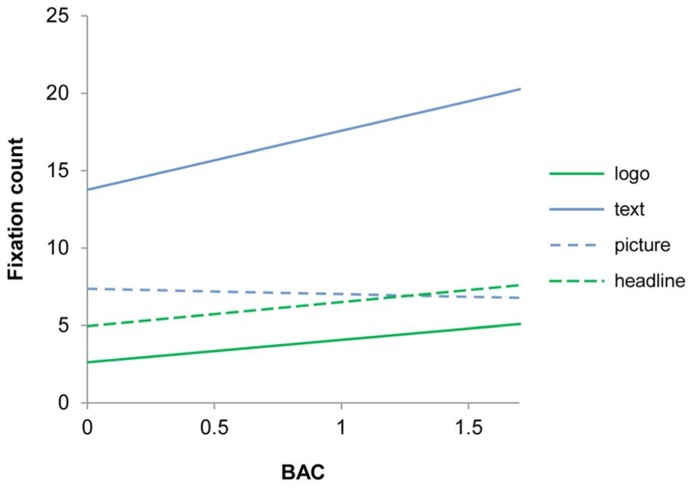
**Effects of blood alcohol concentration on fixation count to ad elements**.

In the second step of the analysis we examined the effect of alcohol intoxication on fixation count to ad elements. Fixation count is the number of times the participant fixates on a stimulus and can be used as an indicator for the strength of interest in a stimulus or as an indicator of confusion. The analysis was carried out by means of a linear mixed model using fixation count as dependent variable and ad element, ad version, brand, and BAC as independent variables. The analysis revealed a significant effect of ad version, *F*(2,875.55) = 5.42, *p* < 0.01, a significant effect of brand, *F*(11,875.73) = 3.44, *p* < 0.01, a significant effect of ad element, *F*(3,880.09) = 31.08, *p* < 0.01, and a significant interaction effect between BAC and ad element, *F*(3,881.79) = 2.88, *p* < 0.05. The interaction effect between BAC and ad element is illustrated in **Figure 2**.

It is clear from **Figure 2** that alcohol intoxication does not have any effects on fixation count except for the text element for which fixation count increases as a function of BAC. However, the effect of alcohol intoxication on fixation count to the text element does not necessarily mean that intoxicated participants read more of the ad copy relative to sober participants. An alternative and perhaps more plausible interpretation is that alcohol intoxication has a detrimental effect on reading abilities which necessitates more fixations to process the same amount of text.

In the last step of the analysis we examined the effect of alcohol intoxication on brand recall. The analysis was carried out by means of a generalized estimating equation with a logit link function and a binomial response distribution using ad recall as dependent variable and BAC and ad attention capture as independent variables. The results are reported in **Table 5**.

**Table 5:** Effect of alcohol intoxication on brand recall controlling for ad attention capture.

	B	Wald χ^2^	df	Significance	Goodness-of-fit
**Model 1**
Intercept	-0.42	8.26	1	0.000	QICC = 1349
BAC	-0.72	14.10	1	0.000	
**Model 2**
Intercept	-0.31	4.62	1	0.030	QICC = 1328
BAC	-0.64	11.72	1	0.000	
Fixated (no)	-0.86	19.48	1	0.000	
Fixated (yes)	0				

The analysis revealed that alcohol intoxication has a significant negative effect on brand recall (Model 1) although the effect diminishes when controlling for ad attention capture (Model 2). Having shown in step 1 of the analysis that alcohol intoxication has a significant negative effect on attention capture for the entire ad, it should be clear that the negative effect of alcohol intoxication on brand recall is partially mediated by attention capture. In other words, alcohol intoxication significantly diminishes brand recall, but only a part of this effect is due to memory loss another part is due to reduced ad attention capture.

In more popular terms consuming one or two beers diminishes the probability of brand recall from 40 to 36% while being heavily intoxicated further diminishes the probability of brand recall to 17%.

### DISCUSSION

Study 2 examined the effects of alcohol intoxication on attention to advertising under a voluntary exposure paradigm which, compared to Study 1, more realistically simulates real world ad exposure. The results suggest that alcohol intoxication changes attention to ads in several ways. First of all, alcohol intoxication lowers the likelihood of participants fixating on the ad and particularly the logo. This result is in stark contrast to Study 1 showing an increase in the salience of logos as a result of alcohol intoxication. Since the experimental stimuli were identical for the two studies this can only mean that alcohol intoxication lead to remarkably different effects on attention under a voluntary versus a forced exposure paradigm. Another interesting finding in Study 2 was that text elements received more fixations under alcohol intoxication. This, however, does not mean that intoxicated participants read more of the text than sober participants. More likely, intoxicated participants need additional fixations to process the same amount of text due to impairments in conceptual processing. Most importantly, Study 2 showed that alcohol intoxication has a strong negative effect on brand recall even when controlling for ad attention capture. The analysis also revealed that the detrimental effect of alcohol intoxication on brand recall is partially mediated by the diminished attention capture.

## GENERAL DISCUSSION

### SUMMARY OF FINDINGS

Study 1 found that alcohol intoxication leads to a significant increase in the visual salience of logos compared to other ad elements. The result is in line with psychopharmacological findings on the effect of alcohol intoxication on cognitive processing. The increased salience of logos occurred regardless of the ad composition and did not affect other perceptual ad elements like pictorial representations. However, these results were obtained in a forced exposure paradigm and we decided to conduct a second experiment to assess the effect of alcohol intoxication in a more realistic voluntary exposure paradigm.

Study 2 examined attention to ads in a voluntary exposure paradigm and found that alcohol intoxication had a negative effect on fixation likelihood for the ad as a whole and for each individual add element. While diminishing the overall attention capture for all ad elements alcohol intoxication increased the number of fixations to the text element which could suggest that intoxicated participants needed more fixations in order to process the text. Furthermore, alcohol intoxication had a strong negative impact on subsequent brand recall which means that a high degree of alcohol intoxication diminishes brand recall by more than 50%.

Interestingly, the two studies demonstrate that alcohol intoxication can lead to dramatically different outcomes depending on whether ad exposure is forced or voluntary. In the forced exposure condition alcohol intoxication increases the salience of the logos which is generally beneficial for the advertiser. However, in the voluntary exposure condition alcohol intoxication leads to a significant decrease in the overall attention to ad elements which is detrimental the effectiveness of advertising.

### MANAGERIAL AND POLICY IMPLICATIONS

From a managerial perspective our results lead us to the conclusion that advertising under licensed premises should constrain itself to the use of reminder ads intended to increase the accessibility of the brand or product. The main reason for this suggestion is that alcohol intoxication impairs conceptual processing of ads which limits the probabilities of persuasion through central route argumentation (Petty and Cacioppo, 1986). It also appears that alcohol intoxication increases the visual salience of logos but only under forced exposure. Under a voluntary exposure paradigm with many visual distractors (in this case magazine articles) alcohol intoxication actually diminishes the likelihood of fixating the ad and the logo. One consideration for advertising under licensed premises would therefore be the degree to which one can control distractors in the environment. One clever strategy which has become popular in many bars is placing ads directly above urinals which, one could argue, is as close as one can get to forced exposure.

Another consideration is that the advertised product should furthermore be for immediate consumption since brand recall will diminish considerably as a function of alcohol intoxication. Using advertising under licensed premises for consumer learning of for instance new products would therefore have to consider the extra expenditure to reach the same degree of consumer learning.

From an ethical perspective the present research solves one issue but raises another. Importantly, there were no indications that alcohol intoxication led to extra influences of advertising on consumers. On the contrary, alcohol intoxication was found to impair conceptual processing of ads as well as recall for the advertised brands which necessarily lowers the effects of persuasion attempts. On the other hand, it was demonstrated that alcohol intoxication under some conditions increases the visual salience of logos which could be used for increasing the accessibility of products for immediate consumption. This could be problematic if advertising under licensed premises for products like alcohol or cigarettes lead to an increased consumption of these products, but the enhanced impact idea could also be used for advertising of cab services or protection against sexually transmitted diseases. The issue is particularly important since other studies have suggested that intoxicated people respond stronger than sober people both to irresponsible short-term incentives as well as more prudent long-term goals (MacDonald et al., 2000).

### LIMITATIONS AND FUTURE RESEARCH

One of the main limitations to our studies is the fact that all data collection took place in a lab environment. This naturally limits the external validity of the results and an important step for future research would therefore be to study attention to ads in more natural environments.

Another important limitation in both studies stems from the decision to measure rather than manipulate the BAC. Our decision to measure BAC rather than manipulate it was based on ethical considerations. However, choosing this approach we had to contend with the fact that participants were not randomly assigned to experimental conditions. It is easy to imagine that some participants are more likely to engage in alcohol consumption and that this tendency could be correlated with other traits that could have influenced the experimental results. Furthermore, because participants were recruited in a student club it was impossible to control for exposure to nicotine which has been shown to influence attention (Bekker et al., 2005). Future research should ideally take these issues into consideration in designing experiments both aiming for high external validity using methods such as mobile eye tracking yet avoiding threats to internal validity such as possibly non-random assignment of participants to experimental conditions and control over exposure to other stimulants.

## Conflict of Interest Statement

The authors declare that the research was conducted in the absence of any commercial or financial relationships that could be construed as a potential conflict of interest.

## AUTHOR NOTE

Parts of this article are taken from abstracts presented in the 39th EMAC Conference (Jeppesen and Scholderer, 2010) and the Conference on APA Convention (Orquin et al., 2013b).

## References

[B1] Bekker E. Böcker K. Van Hunsel F. Van Den Berg M. Kenemans J. (2005). Acute effects of nicotine on attention and response inhibition. *Pharmacol. Biochem. Behav.* 82 539–548 10.1016/j.pbb.2005.10.009 16360813

[B2] De Cesarei A. Codispoti M. Schupp H. T. Stegagno L. (2006). Selectively attending to natural scenes after alcohol consumption: an ERP analysis. *Biol. psychol.* 72 35–45 10.1016/j.biopsycho.2005.06.009 16157440

[B3] Hashtroudi S. Parker E. S. DeLisi L. E. Wyatt R. J. (1983). On elaboration and alcohol. *J. Verbal Learn. Verbal Behav.* 22 164–173 10.1016/S0022-5371(83)90123-8

[B4] Jensen B. B. Orquin J. L. Bech-Larsen T. (2014). What distinguishes passive recipients from active decliners of sales flyers? *J. Retailing Consum. Serv.* 21 1–8 10.1016/j.jretconser.2013.07.008

[B5] Jeppesen H. B. Scholderer J. (2010). Visual attention to advertising under the influence of alcohol. *Paper Presented at the 39th EMAC Conference* Copenhagen

[B6] MacDonald T. K. Fong G. T. Zanna M. P. Martineau A. M. (2000). Alcohol myopia and condom use: can alcohol intoxication be associated with more prudent behavior? *J. Pers. Soc. Psychol.* 78 605 10.1037/0022-3514.78.4.605 10794369

[B7] Maylor E. A. Rabbitt P. Sahgal A. Wright C. (1987). Effects of alcohol on speed and accuracy in choice reaction time and visual search. *Acta Psychol.* 65 147–163 10.1016/0001-6918(87)90024-2 3687476

[B8] Orquin J. L. Bagger M. P Mueller Loose S. (2013a). Learning affects top down and bottom up modulation of eye movements in decision making. *Judgm. Decis. Mak.* 8 700–716

[B9] Orquin J. L. Jeppesen H. B. Scholderer J. Haugtvedt C. P. (2013b). Advertising attention capture and memory for brands under alcohol intoxication. *Paper Presented at the APA 121st Annual Convention* Honolulu

[B10] Orquin J. L Mueller Loose S. (2013). Attention and choice: a review on eye movements in decision making. *Acta Psychol.* 144 190–206 10.1016/j.actpsy.2013.06.003 23845447

[B11] Orquin J. L. Scholderer J. (2011). Attention to health cues on product packages. *J. Eyetracking Vis. Cogn. Emot.* 1 59–63

[B12] Peschel A. O. Orquin J. L. (2013). A review of the findings and theories on surface size effects on visual attention. *Front. Psychol.* 4:902 10.3389/fpsyg.2013.00902 PMC3856423 24367343

[B13] Petty R. E. Cacioppo J. T. (1986). The elaboration likelihood model of persuasion. *Adv. Exp. Soc. Psychol.* 19 123–205 10.1016/S0065-2601(08)60214-2

[B14] Pickworth W. B. Rohrer M. S. Fant R. V. (1997). Effects of abused drugs on psychomotor performance. *Exp. Clin. Psychopharmacol.* 5 235 10.1037/1064-1297.5.3.235 9260070

[B15] Pieters R. Warlop L. Wedel M. (2002). Breaking through the clutter: benefits of advertisement originality and familiarity for brand attention and memory. *Manag. Sci.* 48 765–781 10.1287/mnsc.48.6.765.192

[B16] Pieters R. Wedel M. (2004). Attention capture and transfer in advertising: brand, pictorial, and text-size effects. *J. Mark.* 68 36–50 10.1509/jmkg.68.2.36.27794

[B17] Pieters R. Wedel M. Zhang J. (2007). Optimal feature advertising design under competitive clutter. *Manag. Sci.* 53 1815 10.1287/mnsc.1070.0732

[B18] Saults J. S. Cowan N. Sher K. J. Moreno M. V. (2007). Differential effects of alcohol on working memory: distinguishing multiple processes. *Exp. Clin. Psychopharmacol.* 15 576 10.1037/1064-1297.15.6.576 PMC2658822 18179311

[B19] Söderlund H. Grady C. L. Easdon C. Tulving E. (2007). Acute effects of alcohol on neural correlates of episodic memory encoding. *Neuroimage* 35 928–939 10.1016/j.neuroimage.2006.12.024 17303439

[B20] Wedel M. Pieters R. (2007). A review of eye-tracking research in marketing. *Rev. Mark. Res.* 4 123–147

